# Social Resistance and Spatial Knowledge: Protest Against Cruise Ships in Venice

**DOI:** 10.1007/s00048-022-00340-z

**Published:** 2022-08-02

**Authors:** Janine Schemmer

**Affiliations:** University of Klagenfurt, Klagenfurt, Austria

**Keywords:** Cruise industry, Spatialization, Infrastructure, Protest, Spatial knowledge, Transnational networking, Kreuzfahrtindustrie, Verräumlichung, Infrastruktur, Protest, Räumliches Wissen, Transnationale Vernetzung

## Abstract

Cruise ships are at the same time among the most popular and most controversial means of travel. Photos of oversized ships, passing through the historic center of Venice, have become iconic. This paper explores the background of the debate over cruise ships in Venice. Using research at the intersection of culture and technology, the history of technology, urban anthropology, and social movement theory, it sheds light on how the spatialization of the cruise industry through infrastructures affects Venice and the lagoon. In this paper, I will retrace the development of these interdependencies to show how activists, associations, and citizen campaigners address and perform these entanglements. Protest has turned the ship into a powerful symbol for the infrastructural appropriation and transformation of natural and urban space. Since transportation and traffic routes influence people’s everyday lives, it is important to consider their impacts on practices, spaces, and relations, especially in a city like Venice, where footpaths and waterways form an important element of the identity of both the city and its inhabitants. Through its actions, its tacit knowledge of local space, and the explicit knowledge the protest network produces, it both opposes and adds to hegemonic discourses. I argue that the cruise ship has been transformed into a metaphor of global capitalism, which in turn renders it a symbol with transnational impact.

## Abstract (de)

Kreuzfahrtschiffe zählen gegenwärtig zu den beliebtesten und zugleich umstrittensten Verkehrsmitteln. Das überdimensionale Schiff vor der Kulisse des historischen Zentrums von Venedig wurde zu einem ikonischen Bild. Der Artikel beleuchtet den Hintergrund dieser Debatte und untersucht die Verräumlichung der Kreuzfahrtindustrie durch Infrastrukturen und ihre Auswirkungen auf Venedig und die Lagune aus kulturanalytischer Sicht, und nimmt die Schnittmenge von kulturwissenschaftlicher Technikforschung und Technikgeschichte, kulturwissenschaftlicher Stadtforschung und der Analyse sozialer Bewegungen in den Blick.

In meinem Beitrag zeichne ich die Entwicklung dieser Zusammenhänge nach und zeige, wie Aktivist:innen, Vereine und engagierte Bürger:innen diese Verflechtungen aufgreifen und performen. Der Protest machte das Schiff zu einem wirkmächtigen Symbol für die infrastrukturelle Aneignung und Umgestaltung natürlicher und städtischer Räume. Da Transport- und Verkehrswege stets den Alltag der Menschen beeinflussen, gilt es, ihre Auswirkungen auf Praktiken, Räume und Beziehungen zu berücksichtigen, besonders in einer Stadt wie Venedig, wo Fuß- und Wasserwege stark zur Identität der Stadt und ihrer Bewohner:innen beitragen. Durch die Aktionen, das implizite Wissen über lokale Räume und das explizite Wissen, das das Protestnetzwerk produziert, widersetzt es sich hegemonialen Diskursen und trägt zugleich zu diesen bei. Ich argumentiere, dass das Schiff zu einer Metapher des globalen Kapitalismus wurde und daher als Symbol mit transnationaler Wirkung fungiert.

## Mobilising Against Cruise Ships—An Introduction

Immense floating boxes pass through the basin of San Marco: they are white, they call them ships, and as a matter of fact they should be, but compared to the splendid ships of the past […] their only function is to carry passengers, as many as possible.

These ships have neither refinement nor good taste, they are inspired by the casinos of Las Vegas, and on board they keep what they promise: a resort-like holiday, […] guests in sandals and shorts, oozing with sun cream. Cruise passengers who are part of the perhaps 30 or more million visitors a year that suffocate Venice, increasingly turning it into a kitsch postcard of itself, because the Stazione Marittima is by now one of the main gateways to the kind of “hit and run” tourism that the authorities only in words say they want to oppose to. […] Many Venetians no longer want them and are mobilizing […] but large ships cannot be banned just because they are ugly or disgraceful. They are actually rather harmful and dangerous for the city and its people, despite the Port Authority’s claims to the contrary. (Testa 2011: 5f.)

These are the opening lines of “And they call them ships,”^1^ a pamphlet written by the Venetian journalist Silvio Testa. The publication, which was one of the first to discuss the impact of cruise ships on the lagoon city, was the product of both Testa’s work as a journalist and his involvement in local protests. He used to be the spokesperson of the Committee No Big Ships—Lagoon Common Good (*Comitato No Grandi Navi—Laguna Bene Comune*).^2^ Since 2012, the committee has been one of the main actors in protests against the presence of cruise ships in Venice’s historical center and its lagoon. In this short passage, Testa draws on issues that have become central discourses in international debates about cruise ships. He emphasizes their disproportionate size and mocks the mode of travelling as a sea voyage characterized by aimlessness (Scheppe 2009: 304). By referring to the *Stazione Marittima*, he hints at the spatial and functional transformation of a former focal point of Venice: once the trading hub and commercial heart, it was transformed into an area whose sole function is the processing of thousands of passengers. With many inhabitants of the city protesting the authorities’ lack of action and the Port Authority’s disregard for their concerns, he identifies central issues surrounding the cruise industry, with governing bodies and inhabitants holding opposing views concerning issues of pollution, erosion, and sustainability.

Cruise ships are at the same time among the most popular and most controversial means of travel. Among European destinations, reports highlighting the effects of the cruise industry are largely limited to Venice, mainly due to its character as a city that cultural representations have for centuries described as a place in decline. The oversized ship in the historic center of Venice has become an iconic image. While the gondola is emblematic of the romanticized Venice of regular tourism, the cruise ship has become the symbol of the drastic impacts that mass tourism can have (Mordue 2017; Zanardi 2017). Many articles, reports, and documentaries (The Venice principle 2012)^3^ have examined aspects of the city’s condition as a place that is suffering from excessive tourism while at the same time losing its population.

Conflicts surrounding cruise ships, which have also taken hold in other cities, provide the starting point of this article. Although Venice dominates media representations concerning the destructive impact of cruise ships, it can stand in for the situation in several other port cities in the Adriatic and Mediterranean. Local debates tend to focus on future routes and passenger terminals, hence on the infrastructures of the cruise industry which transform and shape the natural environment, urban space, and everyday life. The example of Venice shows that the cruise industry, and the maritime economy more broadly, unbalance spatial orders and urban practices (Schemmer 2020). This article argues that the cruise ship symbolizes this mechanism. In my contribution, I will approach the issue by analyzing the spatialization of the industry through its infrastructures, its spatial effects, and through the ways inhabitants interact with them.^4^ The article examines the ways in which the *Comitato No Grandi Navi* engages with the cruise industry and its infrastructures to counter the spatialization of the sector. I will show how the network acquires counter- and extra-expertise and shares, spreads, and applies its spatial knowledge to reappropriate space and reclaim a say in the process.

The movement *Comitato No Grandi Navi* has taken on a central role as an active agent of urban change. In its symbolic actions, it conspicuously blocks the passageways used by cruise ships and casts them as transnational symbols of profound ecological, economic, social, and cultural imbalances. The Venice protests attracted significant media attention both in Europe and the rest of the world.^5^ A heterogeneous network of activists, associations, and citizen campaigners, united in their mobilization against the cruise industry in Venice and the lagoon, has expressed its dissent toward the implementation of infrastructures which exclusively support tourism and thereby subject large parts of urban space and urban governance to tourist needs. By connecting their spatial knowledge to contested infrastructural developments, they have become an important voice on cruise tourism in Venice. I will discuss the ways in which protesters share, spread, and apply their knowledge to reappropriate these spaces and reclaim their voice. Yet the structures surrounding the cruise ship are more complex than the dominant discourse on big ships suggests. Venice is not only a central destination for cruise tourism, but Marghera, the industrial part of the lagoon located on the mainland, is also home to one of the production sites of the Italian shipbuilding company Fincantieri, which has been one of the world leaders in the manufacture of cruise ships since the 1980s. In the public perception, however, Marghera and its manufacturing capabilities remain invisible.^6^ Thus, the local field of tension is situated between the limits of popular discourses about the city, the spatialization of the industry, and the governance of these processes. In my contribution, I want to retrace these interdependences, depict the production and circulation of knowledge by the network, shed light on how protests address and perform these entanglements, and analyze the counter-narrative the *Comitato* initiated to challenge hegemonic political accounts and actions through the symbol of the big ship.

This is where this paper ties in with current debates about infrastructure projects and the demand for social participation, and where it reveals the transnational elements of infrastructure studies. Cultural analysis of infrastructures focuses on the connections between the tangible and intangible dimensions of artifacts and technical systems, as well as their social and cultural effects (Strang 2016; Flitner, Lossau, Müller 2017). The concept of infrastructure also includes power constellations (van Laak 2001) and can be understood as “a mixture of political rationality, administrative techniques, and material systems” (Larkin 2013: 331). Dirk van Laak highlights the symbolic value of infrastructures and emphasizes their significance as “lifelines of a community” (2018: 69). However, this raises the question of who belongs to a certain community and who has a stake in infrastructure projects. Hence, analyzing the “processes of infrastructurisation focuses decidedly on the political, economic and social decisions that are inscribed in infrastructure” (Niewöhner 2015: 492), and it shifts the focal point from the artifact to the “everyday translation processes between materiality and normativity” (Ibid.). To grasp the structures and actors involved in protests against cruise infrastructures, I draw on the concept of relational infrastructure that sheds light on the “relation to organized practices” (Star & Ruhleder 1996: 113) by focusing on the connection between the system and the actors involved. A relational approach to infrastructures emphasizes the linkages “between formalized knowledge, social interaction and organizational structure” (Niewöhner 2014: 343), and touches upon the tacit knowledge of individuals or groups and their practices.

Infrastructures challenge spatial as well as social orders (Sheller 2009) and evoke tensions that manifest themselves in practices of resistance. The entanglements between technical, political, and social fields can be seen in the case of Venice’s cruise protests. The concept of spatialization can be used to contextualize the power relations that manifest themselves in this process. Spatialization makes space its central focus and uses it to show the relationship between knowledge, power, discourse, and representation, which is also connected to the question of identity formation (cf. Kajetzke/Schroer 2013: 10). Social conditions are constructed, shaped, and expressed in space. Space establishes order but is also characterized by action (Lefebvre 1991). Therefore, spatialization takes into account not only material dimensions and practices but also the ways in which these are discursively negotiated. My ethnographic research draws on participant observation during regular visits to Venice, analyses of press coverage and gray literature from the protest network, and informal conversations and interviews with activists, citizen campaigners, and the local population more broadly.

In what follows, I will introduce the central protagonists in the protests against big ships in Venice. I will then reflect on the cruise ship as a metaphor for a global capitalism that subordinates people, urban space, and the environment to economic logic. This is also where the transnational dimension of social resistance against cruise infrastructures and tourism is highlighted, as it has clearly European scope. Since it is important to examine these conflicts in their historical dimension, I will then outline the local development and draw attention to the effects of contested cruise infrastructures, which deeply influence structures and rhythms on water as well as on land. Finally, I will focus on direct action by the committee, its embodied knowledge, and the transnational relations it established.

## No Grandi Navi—Fighting *Naval Gigantism* with Spatial Knowledge

The *Comitato No Grandi Navi* addresses the ecological and social issues connected to cruise tourism and speaks out against the authorities governing the relevant industries. The movement was established as a network of exchange, knowledge production, and knowledge distribution. It mobilizes individuals and groups to act on site. Its actions are directed at the ship as an oversized object out of proportion to the places it visits. In what follows, I will describe the structure of the movement, its aims, and its social composition.

*Comitato No Grandi Navi* started out as a neighborhood information platform about big ships. It was turned into a civic protest group after the Costa Concordia accident in January 2012, at the Isola del Giglio in Tuscany, where 32 people died. The fear of similar accidents in Venice due to ever larger ships, as well as concerns over the management of big infrastructure projects in this delicate urban environment, brought several activist groups together. In 2012, they started their joint initiative against cruise ships in the lagoon and the city. The *Comitato* is organized as a collective actor (Buechler 2000: 149), with individual actors connected on a local level. This open structure is a typical feature of recent social movements: “[…] independent of specific milieus, subcultures and social affiliations, the most recent forms of protest are characterized by network-like coordination and non-identitarian forms of participation within the framework of little formalized, temporarily limited structures” (Balint et al. 2014: 10). Characterized by a collective approach, the *Comitato* brings together individuals, associations, and groups (Della Porta & Diani 2006) that had already been established at the local level. Several members and friends of the committee have been involved in local politics, have work experience in the industrial sector, or have expertise in dealing with government authorities. Members of the committee empower themselves by acquiring knowledge and establishing counter-expertise. When the construction of the flood barrier project *MOSE*—short for experimental electromechanical module (*modulo sperimentale elettromeccanico*)—began in 2002, the Committee Save Venice with the Lagoon (*Comitato Salvare Venezia con la Laguna*) was founded. Since then, it has organized protests, produced informational documents, and appealed to local and national authorities. Since 2005, the Permanent Assembly NOMOSE (*Assemblea Permanente NOMOSE*) has followed and commented on the structure’s implementation. The association EnvironmentVenice (*AmbienteVenezia*) was created in 2007 to advocate for the protection of the lagoon, the coastal system, and the rights of the local population.

The *Comitato No Grandi Navi* is characterized by a strongly transversal character in its organization, its communication strategies, and its actions. Combining different approaches, materials, and modes of action, the network has established a “strategic cooperation around an objective beyond the scope of which, each associated member maintains its specificity” (Vianello 2013: 9). The heterogeneous composition of the committee reflects the variety of fields it engages in, and it visibly affects its production of counter- and extra-expertise. Since the committee has no official spokesperson, several persons held leadership positions over the years. Activists from the antifascist social center Occupied Laboratory Morion (*Laboratorio Occupato Morion*) play a crucial role, with Tommaso Cacciari being an important representative of the committee. Aside from assemblies, talks, and informational meetings, the activists organize and coordinate direct actions connected to the protests in the city. *Laboratorio Occupato Morion* stands in the tradition of the social movements of the 1970s, with “strategies of struggle, discourses and actions of the radical approach to contestation through protest” (Vianello 2013: 9). However, its high visibility has not necessarily gained it the support of the wider public (Kurik 2016), as some interviewees, including members and non-members of the committee, have pointed out.^7^ Yet despite divergent political orientations or disagreements over appropriate protest tactics, all interviewees shared the group’s general critique of local politics. *AmbienteVenezia* plays another important role in the committee. Its spokesman, Luciano Mazzolin, a former petro-chemical worker and councilor of the Province of Venice, is another of the committee’s spokespersons. Through the collection and circulation of documents, *AmbienteVenezia* provides a record of incidents in the lagoon and on mainland Venice. It also runs a continuous information campaign on industrial, ecological, and political developments, and makes this information public through different channels. Aside from these central actors, there are experts from technical and academic fields who participate in the *Comitato, *such as Giuseppe Tattara, former professor of economics at the Università Ca’ Foscari, and the architect and writer Gianni Fabbri, who contribute in-depth analyses and reports on the ecological and social effects of the cruise industry on urban development (e.g. Fabbri & Tattara 2014). Other members and supporters commit by producing or collecting and spreading a large variety of documents, ranging from reports to letters. A central platform for the distribution of information and the coordination of action is a mailing list, where synopses of press coverage are regularly circulated. In addition, the occupied S.a.L.E. Docks, a space for critical artistic expression, has become an important site for the movement and regarding the debate. Another initiative which cooperates with the committee is the non-profit association *We are here Venice*, which has strong transnational connections and specifically addresses ecological challenges as well as questions of policymaking in Mediterranean ports.

The committee has acquired extensive extra-expertise, which it uses to establish counter-narratives to those of the governing authorities. Each group contributes its specific knowledge and experts to the committee. The committee then spreads the word about its plans and actions via several (social media) channels. This in turn results in new connections to other movements, such as the local section of the global movement *Fridays for Future*. Furthermore, different participants perform different forms of protest. Members and friends of the committee differ regarding age, expertise, and social background. The committee brings together left activists, civic associations, and members of the local community. This alliance illustrates the complex local constellations and long-lasting commitments of many actors and groups. It also emphasizes the tight interrelation and cross-border resonance of ecological and social issues.

Tourism dominates not only the popular imagination about Venice but also its local politics. The city’s inhabitants have been experiencing the subordination of local social structures to tourist needs for decades, a process that has led to displacement through the establishment of a tourist monoculture. While the historic center had 174,808 inhabitants in 1951, the figure for 2014 was 56,311 (Casagrande 2016: 125). Although tourism has long been a lucrative business for investors and residents alike, it is mainly the latter who have addressed the industry’s negative effects on the city over the last two decades. These effects have driven the city’s inhabitants to the streets and squares in increasing numbers. Coalitions of associations, organizations, and committees are evidence not just of the city’s problems, but also of the initiative and agency of its inhabitants (cf. Araya López 2021; Dlabaja 2021). This engagement underlines Henri Lefebvre’s notion of the city “as a resource of society” (Schmid 2011: 32), and it shows a demand for participation in the shaping of the economic, social, and cultural resources which are increasingly being transferred to external stakeholders.

**Fig. 1:**
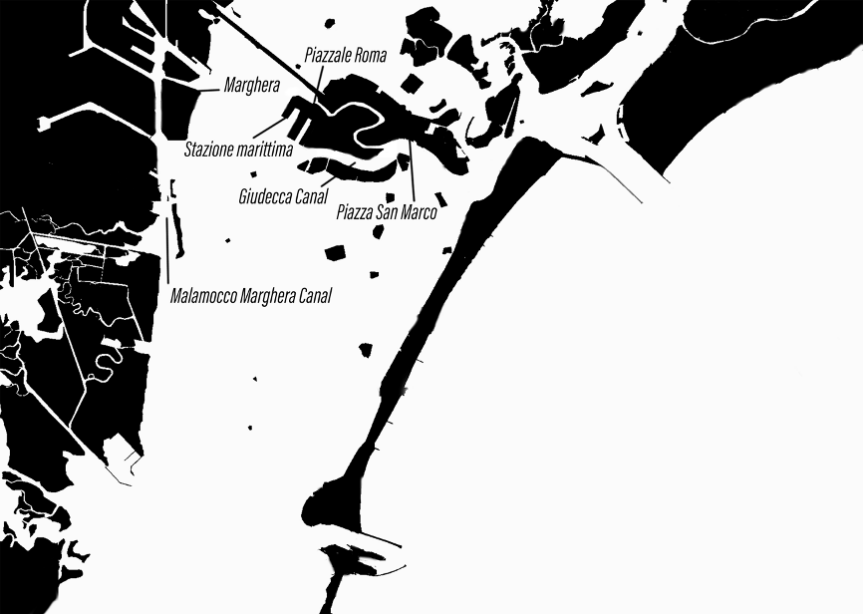
Simplified map of Venice, lagoon and mainland Venice (picture by Janine Schemmer)

The demand for an urban policy that focuses on the needs of the inhabitants is one that several local initiatives share with the *Comitato No Grandi Navi*. However, the committee does not advocate against the presence of tourists as such. The problems it tries to address concern the political management of the environmental and social consequences of tourism and the Port Authority’s way of governing the shipping industry. The *Comitato *expresses its goals in its slogan, its name, its symbol, and its messages and actions. The slogan is clear and straightforward: “Ships out of the lagoon now and forever” (“*Fuori le navi dalla laguna ora e sempre*”). It also reinforces this idea visually. Its distinctive symbol, a crossed-out ship, communicates the demand to ban big ships. In its diagnostic and prognostic framing, the committee explicitly positions itself against big ships in the present as well as the future. In its argumentation, *No Grandi Navi* emphasizes the disproportionality of a giant ship against the backdrop of a mid-sized city and its natural and urban environment. The disproportionate and “monstrous dimension” (Mancuso 2009: 156) of the “spaceship of ‘the modern’” (Settis 2014: 110) is one of its central arguments. Above all, it is the route of the ships that has been, and still is, the focus of political mobilization (Fig. 1).

Running right through the Giudecca Canal, one of the main canals passing the historic center and the Piazza San Marco, the waves created by ever more and increasingly larger ships damaged the foundation of the city since years. The emission of sulphur oxides from cruisers contributes to air pollution. Moreover, the ability of ships to enter the lagoon necessitates expansion of waterways and further deepening of existing channels, which in turn could cause additional damage. Such infrastructural adjustments harm the natural environment. The committee aims to convince both local and national policymakers that personal, social, and environmental wellbeing should not be subordinated to economic logic (No Grandi Navi 2021). Through the actions of activists and ordinary citizens, a reappropriation of urban infrastructures and spaces has begun. The newsletters of *AmbienteVenezia* make this clear: “Misguided models of development have produced the environmental and social devastation that everyone can see! Only the awareness and mobilization of citizens can change and improve this situation” (2020).^8^*AmbienteVenezia* regularly accuses policymakers of disregard for social concerns as well as a blindness for objectives other than profit maximization. At the same time, its newsletter reads like a general call to a “protest attitude, commitment and (moral) change of values” (Balint et al. 2014: 10). The heterogeneity of the group members, their networks, their long history of activism on ecological issues, and their utilization of academic and journalistic publications have brought the topic to international public attention—which in turn has increased opportunities for forging transnational connections. Most importantly, their struggles have led to a partial victory. In August 2021, the national government announced that large cruise ships will no longer pass the Giudecca Canal in Venice’s historic center—a ban that applies to ships weighing more than 25,000 tons or measuring more than 35 meters in height or 180 meters in length.

By highlighting the externalities of naval gigantism, *No Grandi Navi* is participating in an international debate about the risks that the growing size of cruise and container ships pose. This is a subject that is connected to several other issues.

## Cruise Ships as Symbols of Imbalance—Transversal and Transnational Dimensions

As an important historical trading hub, the ecological and social consequences of maritime trade and transportation have affected the city of Venice for centuries. With the appearance of motorized boats like the *vaporetti* in 1910, new problems, such as wash waves, emerged (Petri 1986: 63). In the following decades, ships navigating the lagoon and the city’s canals steadily increased in size. The last container ship passed the Piazza San Marco in 2000 (Trevisanato 2015: 20). While the political management of the shipping industry has been criticized by local groups for decades, ships transporting goods rather than people did not trigger the same kind of anger. This points to the fact that the cruise ship is more than just a vehicle used for traversing space. It is also a symbol that gives scope to different meanings.

Cruise liners are subject to economic logics and explicitly aim to create illusions (Settis 2014: 117). Alongside their ports of call, they have long become popular destinations in their own right, with advertisements suggesting designs inspired by places like the Piazza San Marco or Milan’s Cathedral Square (Ahoi 2019: 15).^9^ While cruise ports have experienced significant transformations, the tourism industry relies on the popular imagination in its efforts to attract customers (Saarinen 2004). In reality, the alleged dream journey often stands in stark contrast with the working conditions for on-board staff, which are characterized by deregulation and precarity (Chin 2008). A cruise journey is a global experience that takes place in a secured and regulated manner. Destinations can be experienced at a safe distance, without the need for physical proximity or interaction with local populations. At the same time, ships and passengers take up significant amounts of space. The industry is materialized in infrastructures and tourists and manifested through labor migration and the deterritorialized and stratified aspects of the shipping industry (Sekula 1995; Watts 2014). Hence, industry, ship, and cruise travel create polarized and divided opinions.

On the one hand, the cruise ship represents the values of a powerful industry and the dreams of many travelers. On the other, it symbolizes the concerns and anger of activists and local residents. Through knowledge circulation and direct action, the *Comitato No Grandi Navi *has established the cruise ship as a symbol of the profound and long-standing impact of the maritime economy on the shore. In addition, it has helped connect different discourses (Link 1986: 6) in a transnational manner. While current debates over social challenges revolve around concepts like growth, de-growth, and post-growth, the cruise ship reflects the increase of growth through its very dimensions. Hence, it has become a projection screen for different actors and is perceived at the same time as both desirable and destructive. A particularly important issue represented by the symbol of the cruise ship concerns the effects that the dominance of the tourism industry has on Venice’s natural and urban infrastructures as well as its everyday life. The *Comitato No Grandi Navi* frequently refers to the dominance of external interests and the heteronomy this creates. The power of industries like the shipping sector stands in contrast with a city that is shrinking as well as residents who feel that their opportunities are decreasing. While trade once brought wealth to Venice, experts argue that due to an increasingly externalized supply chain, cruise tourism “brings scant benefit to the port territory” (Fabbri & Tattara 2014: 18). Only a small part of the local population is involved in the sector and benefits from it economically. Meanwhile, many suffer from its consequences: “Cruise tourism at Venice has been presented by the local administration, the Port Authority, Venice Terminal Passeggeri and the trade unions as an opportunity wellnigh free of cost, to swell the income of the city and its jobs. This is far from being the case. The earnings stemming from increased tourist demand need to be downsized since they entail large external diseconomies (…)” (Fabbri & Tattara 2014: 32f.). The ship stands for the excesses of the industry and for urban economic policies that are contrary to the interests of many citizens. As Donatella della Porta and Sidney Tarrow put it, one can observe “a shift in the locus of institutional power” from politics to the market, resulting in “the development of a system of ‘complex internationalism,’ which provides both threats and opportunities to ordinary people, to organized nonstate actors” (2005: 2). This “complex internationalism” is reflected in the cruise industry: it is a symbol of abundance, social injustice, waste of resources, and displacement. In the passage quoted at the beginning, Silvio Testa grapples with this lack of sustainability, and with the profit maximization of an industry that—at least until the outbreak of Covid-19—seemed to represent infinite growth.

The cruise ship puts pressure on the social and political order, and any engagement with it raises the question of who benefits from the industry. Since current debates at times go back several decades, it is crucial to consider contemporary conflicts in their historical dimensions. The ecological and social problems caused by the spatialization of industrial infrastructures in Venice and the lagoon was already present in Italian popular culture in the 1970s, and it has been debated ever since.

## (De)Industrialization, Touristification, and Imagination—Connections Between Island and Mainland

In the media, the destructive impact of tourism on the historic center of Venice usually tends to be the focus of attention. However, the fact that the city is also a production site of cruise ships is rarely discussed. In general, the industrial area of Marghera and the cruise ship industry located there remain largely invisible in the public eye.^10^ An equally blank space in the international perception of the lagoon city is its industrial past. Both of these factors are important for understanding current developments and debates.

The shipping industry shapes the connection between island and mainland in important ways. Its dominance, together with the influence of external interests in local politics (Benevolo 1979), has been subject of debate for years. This imbalance is depicted in the cartoon film “Mr. Rossi in Venice” (1974).^11^ The illustrator, Bruno Bozzetto, comments on societal transformations and points to the complex processes that lie behind seemingly simple actions. The film also hints at the historical dimensions of current debates. It critically portrays clichés about Venice and visualizes the decline of the city, an issue that several historical destinations started to face at the time of its creation. By showing traffic, smog, pollution, frustrated inhabitants, and large-scale infrastructural projects, the short film depicts ecological and political issues that affect the city, its environment, and the people who live and work there. In addition, it addresses the immense influence of consumerism, raising questions about staging, authenticity, power, and the subordination of ecological interests to economic motivations. It is especially noteworthy that the film highlights the tight connection of the island to the mainland part of the city, Mestre, the “unloved child” (Petri 1988: 112) of Venice next to the industrial port of Marghera. Both play a crucial role for the city’s economy and its everyday life. Island and mainland are part of the metropolitan city of Venice that rarely appears in popular representations. This constellation has led to several conflicts concerning the governance of the area as a whole. The division between island and mainland is expressed not only in spatial terms but also in the “island and mainland identity” (Casagrande 2016: 121), which is the subject of heated debate.^12^ The cartoon illustrates the entanglement between island, lagoon, and industrial area through the tentacles of huge passenger and transport ships, reaching out in every direction.

The development of transportation and shipping infrastructures is closely connected to the industrialization and touristification of Venice. In the nineteenth century, the railway and the port enabled the establishment of industries, which remained mainly at the margins of the island and in vicinity to traffic connections (Mancuso 2009: 68), such as the area where the *Stazione Marittima* is located. This former industrial hub today features cruise terminals. To meet growing demand, the Italian government and local politicians started the construction of Marghera on the other side of the lagoon in 1917. This structural transformation led to the relocation of industry from island to mainland. A leading figure in the touristification of Venice was Giuseppe Volpi, who pursued his interests as an industrial entrepreneur, hotelier, and Fascist minister. After 1950, the petrochemical sector became especially important for the development of Marghera. Since the late 1960s, disputes concerning dangerous work conditions and environmental degradation have reinforced the ongoing struggle between safeguarding the historical center on the one hand and expanding industrial development on the other (Zazzara 2017). During the industrial crisis of the 1970s, politics shifted direction, and the island’s value as a tourist destination was rediscovered (Petri 1988: 112). This led to a politically and economically driven deindustrialization and touristification of the island, which took place at the expense of the environment and the residents, and which continues to influence the area to this day.

## Spatialization Through Cruise Infrastructures I—Contested Waterways

Transportation and traffic routes deeply affect people’s everyday lives. Therefore, it is important to consider the “intrinsic relationship between transport and transformation” (Neubert & Schabacher 2013: 25), as well as its impact on mobile and immobile practices, spaces, and relations. The lagoon is both a fragile ecosystem and a central waterway. Due to its environmental problems after decades of industrial use, as well as the divergent interests that are involved in it, it is a highly contested space. Similar to historical developments, current discussions show that traffic routes are not adapted to nature but that nature adapts to traffic routes (Fackler 2006; Mathieu 2007). Although ships of over 25,000 tons do not pass through the Giudecca Canal anymore, the *Comitato* continues its fight to keep cruise ships out of the lagoon. The debate especially revolves around future locations of new cruise terminals and routes for super-size ships. Whether these will be located inside the lagoon or not is a central point of discussion, and the search for a solution dominates daily headlines. In the meantime, the authorities decided on temporary solution by using landing places in Marghera which the *Comitato* calls “a farce”^13^ and regards as unsustainable.

Despite growing criticism, big cruise ships dock in Marghera since April 2022.^14^ Locating the docks in the industrial zone means that cruise passengers debark right next to container terminals. This setting has been heavily criticized as inadequate in terms of safety for the passengers by several actors. Besides, cruise ships pass alongside cargo ships and petrol tankers through the *Malamocco Marghera Canal*, also called *Canale dei Petroli*, dredged in the 1960s to make way for big vessels. Considerations about its enlargement and its opening to passenger transportation go back to at least the 1970s, when the petrol port was enlarged. The canal’s extension was one of the reasons for increased tidal water. Although a special law for safeguarding Venice was passed in 1973 (Mancuso 2009: 109), it arrived too late to prevent environmental damage. In addition, petrochemical plants at Marghera caused health hazards as toxic waste was dumped into the lagoon. And since the waste settles on the seafloor, experts warn against dredging operations. Highly debated and under construction since 2003, the MOSE barrier is meant to prevent flooding (Mancuso 2009: 135). Current political debates focus on the compatibility between the operation of the tidal gates and large ships. In this regard, the MOSE is another contested infrastructure project, since according to its critics its costs outweigh its benefits (Casagrande 2016: 127).

In the context of infrastructural transformations, the future of Marghera is up for debate. New passenger terminals located in this area could also affect the work performed there. Aside from ecological challenges, the construction of a new passenger terminal in Marghera is in conflict with the aims of the metal industry that operates there. In a press release, local representatives of the Italian Federation of Metal Workers (*Fiom*) declared:

It is possible to combine the environmental sustainability of Venice with development and the ability to create new jobs in the area, provided that we stop squandering huge public resources on pharaonic projects designed primarily to benefit tourist lobbies and those economic and speculative powers that have impoverished and emptied the historic city center and destroyed economic and production activities historically linked to the city and its industrial and manufacturing vocation. (Fiom Venezia 2018).^15^

The syndicate presents arguments similar to those put forward by the committee against enormous infrastructural projects, and it emphasizes the importance of diversified economic activities. Likely also due to the fear that tourism might eventually replace the metal industry, they stand against the idea of creating tourist facilities in the industrial port area. This reflects the displacement effects that cruise infrastructures and tourist monocultures have on both sides of the lagoon. In general, the gap between environmental protection and concerns over employment is widening. The *Comitato* emphasizes that it aims to preserve jobs connected to the port industry and that it is engaging with workers themselves. At the same time, it remains firm in its goal to limit the industry’s development. Regarding the planning of terminals, the network argues that “if the alternative is Marghera, we will organise the upcoming protests along the Canale dei Petroli.”^16^ Hence, the committee continues to organize site-specific forms of action explicitly directed at cruise infrastructures.

## Spatialization Through Cruise Infrastructures II—Challenging Urban Rhythms

**Fig. 2:**
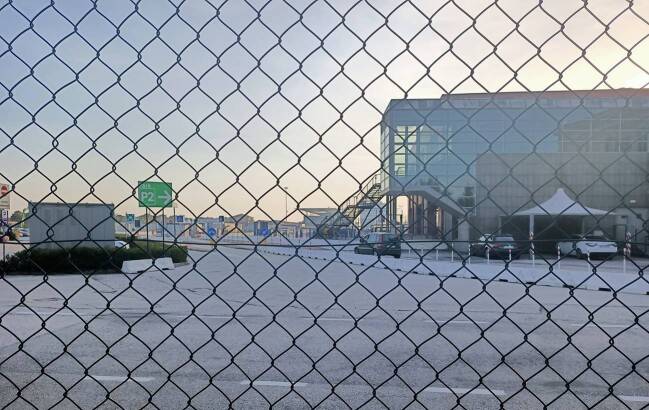
One of the terminals of the *Stazione Marittima*, former central cruise terminal, 2020 ( photo by Janine Schemmer)

With the arrival of cruise tourism, business returned to the island. It appears in the moving ships, their routes, and the terminals that serve them. However, infrastructures also affect cognitive and physical perceptions, which form a substantial part of everyday organization and routine (Star 1999: 380). Therefore, while cruise infrastructures make people move, they also interact with infrastructures of the everyday and with symbolic places of the city, like the *Stazione Marittima, *the former commercial heart of the island. There, the Venice Passenger Terminal (VTP) was founded by the Port of Venice in 1997, whose expansion has been planned ever since. Seven former warehouses were restructured and a number of terminals were newly constructed. The *Marittima* can accommodate up to nine vessels at a time, and the cruise season has been steadily expanding. The Covid-19 pandemic and specifically the political ban on large ships in August 2021 put an end to this development, and the terminals remain mostly inactive up to now.^17^ Since the area is completely fenced off, the cruise terminals are literally isolated from their urban surroundings and not accessible for anyone. During the years in use, the terminals created the link between the ship and the place it visited. At the same time, the ship remained isolated. This situation created both a visual and material detachment of business from city (Fig. 2).

With the construction of new passenger terminals, the trade port’s heritage was further eroded, and the “rhythmicity of the former trading hub is now marked by the arrivals and departures of cruisers” (Sovrani 2009: 25). The transitory use of a cruise terminal and the standardized rhythms it produces stand in contrast with those of the city, with the natural rhythm of the lagoon, and with “rhythms of social habits” (Stahl 2018: 150). Although the terminals are located at the periphery of the city, the “tentacular” ship (Sovrani 2009) reaches out into different areas of lived urban space, leading to a competition between tourists and residents over the use of the space.

**Fig. 3:**
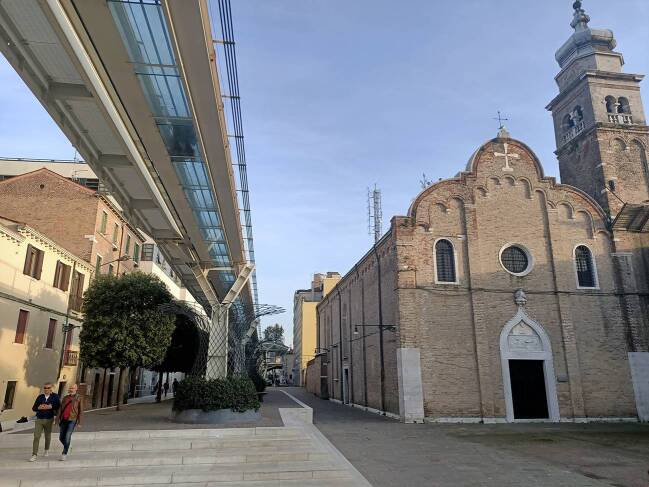
The people mover connecting *Stazione Marittima *to* Piazzale Roma*, 2020 (photo by Janine Schemmer)

An analysis of rhythm allows for the perception of how time and space are interconnected (Lefebvre 2004). Rhythm can be observed in technical facilities, but also in bodily movements. Since 2009, a people mover has connected the VTP with Piazzale Roma, the main traffic junction for accessing the historic center from the mainland. In a three-minute ride, passengers floated over the unmaintained periphery of parking lots and construction sites before they arrived in Piazzale Roma. In a city where people move exclusively on foot or by water, and where distances covered on foot determine the sense of time, a cable railway was installed for the convenience of the cruise tourists—a means of transportation that is emblematic of the passivity and distant closeness often associated with cruise tourism (Fig. 3).

The transitory character of cruise tourists can be seen in their speed. In general, the pace at which tourists move through the city and the velocity with which they consume and appropriate its spaces seem to interfere with the rhythm of everyday life. This is reflected in publications like “When in Venice Walk in Single File: instructions for use of the city” (Berger 2019), which provide visitors with instructions for moving around and actually experiencing it. Cruise tourism amplifies the sensation of a different pace through the concentration of large numbers of people in limited areas for brief periods of time. The flow of people that a docking cruise ship sets in motion differs significantly from that of any other form of tourism, yet their rhythm influences that of the inhabitants considerably. This externally determined timing leads to friction: “When relations of power overcome relations of alliance, when rhythms ‘of the other’ make rhythms ‘of the self’ impossible, then total crisis breaks out, with the deregulation of all compromises” (Lefebvre 2004: 99). This fact is also present in the forms of action taken by the protest network, where the rhythm of the body is vividly represented.

## Resist and Reclaim—Reappropriation Through Embodied Knowledge

**Fig. 4:**
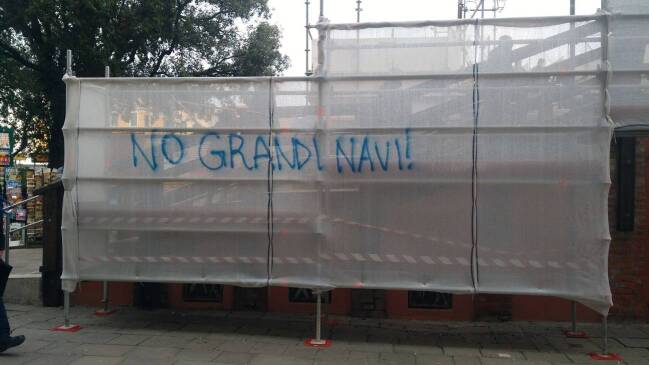
Public space as protest space, 2017 (photo by Janine Schemmer)

When walking Venice’s streets, squares, and bridges, it is impossible to miss the stickers, posters, graffiti, and flags placed throughout the city. Public as well as private spaces have been turned into display areas for the messages of numerous associations and groups. Recent debates have transformed the city into a large protest space, which the residents use to communicate what they see as their right to reappropriate urban space (Fig. 4).

In addition to these visual campaigns, the *Comitato No Grandi Navi* has also engaged in confrontational initiatives over the years. The area around Piazza San Marco, the political center of the former Venetian Republic, is arguably the most symbolically resonant space in the city (Araya López 2021). Despite being Venice’s primary tourist attraction, Piazza San Marco continues to hold symbolic meaning and value for the city’s residents. For them, it represents their former independence. Because of the increase in tourism, as well as the laws that prohibit protests at Piazza San Marco, everyday use of the space by locals has declined. Dominated by tourists and for years framed by passing cruise ships, the piazza has become a symbol of spatial expropriation, an issue that activists address through creative forms of protest. By performing a collective jump into the basin in front of the smaller Piazzetta San Marco, they allude to the issue while also circumventing the ban on protests. The use of their bodies shows their performative and playful approach to this matter, as well as their aim of communicating their right to the space. Their emphasis on embodied and tacit knowledge conveys what it means to live in the city. Like other recent protest movements, such initiatives show a “strong aestheticization […], characterized by symbolic action, such as the symbolic visualization of grievances and the reappropriation of public space” (Balint et al. 2014: 11). The committee has developed creative strategies for appropriating space, which they skillfully use in different locales. They express their spatial knowledge through the use of their bodies and emphasize the cultural significance of symbolic places which otherwise tend to remain unrepresented in the popular imagination. In doing so, they show that both public and political space are constantly renegotiated and produced anew (Fischer-Lichte 2004: 199).^18^

**Fig. 5:**
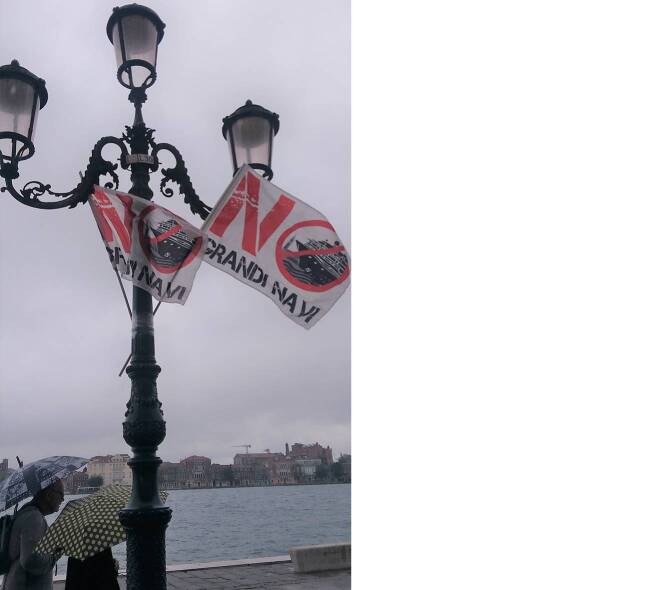
Flags of the *Comitato NoGrandiNavi* during a protest at the *Zattere*, 2017 ( photo by Janine Schemmer)

Spatial competition also occurs on Venice’s waterways, and protests addressing this issue tend to reflect the vernacular meanings that waterscapes have for the city’s inhabitants. Therefore, another important strategic place for direct action has been the *Zattere. *This tongue of land extends about one kilometer along the Giudecca Canal, where cruise ships used to pass by to allow passengers a brief glimpse of Piazza San Marco. In September 2017, a day of protest with the theme “Blockade!” took place along parts of the walkway, with participants installing flags, setting up stands, and providing refreshments (Fig. 5).

The local complaints choir, an established and important instrument of transnational activism, contributed to and amplified the acoustic and auditory dimension of the protest (Donath 2018). On the water, activists turned boats into tools of protest by blocking the passage of ships. By using their row boats to block the movement of big ships, many participants turned their vehicles into effective counter-symbols. Their goal was to disturb the flow of traffic, to delay the arrival of cruise ships by several hours, and thereby to interrupt the system in the same way that it interrupts the paces of the city’s inhabitants. Turning the water into a protest space, activists also drew attention to the fact that waterways are more than just transport routes. They are the streets of the residents and form an important part of the urban space in which people work, spend leisure time, and gather for important events. Regarding their relational dimension, Elizabeth Shove argues that infrastructures should not be perceived “as somehow fixed. Instead, something becomes an ‘infrastructure’ when it stands in an infrastructural role in relation to one or more practices” (Baringhorst et al. 2019: 77). This fluidity manifests itself in urban actions happening in multiple locations.

**Fig. 6:**
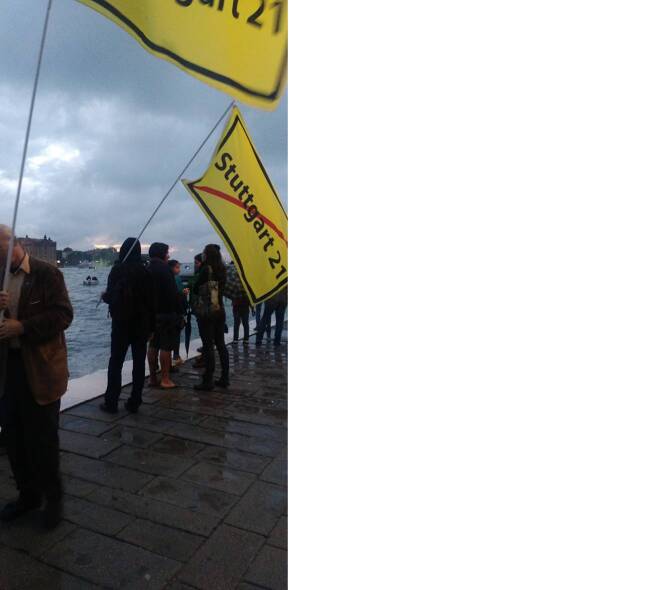
Activists from *Stuttgart* *21* joining protests against big ships at the *Zattere*, 2017 (photo by Janine Schemmer)

These types of protests, performed in symbolic spaces with specific meanings and functions, show that the committee aims to reach the alliances of local and global stakeholders who profit from the industry (Kryst/Zajak 2017: 63). This includes the Port Authority as well as the local and national government. In addition, these protests try to remind cruise tourists of the structural conditions that enable their travels, and to make them aware of the blind spots the latter have. But protests of this kind also function as networking opportunities for their participants. In 2017, protests were explicitly used to create the “European assembly of movements for the defense of territories, for climate justice and democracy.” The assembly took place in the S.a.L.E. Docks and called for a “new European cycle of battles” (No Grandi Navi 2021).^19^ Brochures on display provided information on local and global initiatives. Participants, residents, and activists exchanged views and discussed experiences in workshops and during other activities. On that occasion, I encountered German protesters against the *Stuttgart 21 *project, one of the most controversial German urban development projects in recent history.^20^ Waving their yellow flags, they were easily recognizable amidst the *No Navi* crowd (Fig. 6).

When inquiring about their motivations for joining the initiative, one participant told me that the get-together was about the right to have one’s voice heard. He described such gatherings as connected to broader critiques of big and unnecessary infrastructure projects, whether in places like Stuttgart or in Italian cities. Through common causes, such as the No High Speed Trains (*No Tav*) movement’s fight against highspeed railways in the Susa Valley in Piedmont, they got to know the committee.^21^ The *Stuttgart 21* protests touch on “the question of power in representative democracies, the use of economic resources and, more generally, the question of the right life” (Schönberger 2014: 28). *AmbienteVenezia* comments in a similarly critical way on the possibility of a future-oriented coexistence. “This is the city and the territory we want” (“*Ecco la citta’ e il territorio che vogliamo*”) reads their newsletter, which they use to describe risks and provide alternatives to current government plans. All of these movements share the conviction that the political “no longer constitutes a claim to power, to hegemony” (Schönberger 2014: 28), proposing instead a collaborative approach to knowledge production and governance.

Other demonstrations address strictly local topics and are explicitly directed at the city administration or the Port Authority. On June 10, 2018, the committee organized a “Protest March for the Dignity of the City.” The protest turned into a substantial campaign, bringing together more than 70 urban committees, associations, trade unions, parties, and individual citizens. On their banners, participants called for an urban policy focused on the needs of the city’s inhabitants. They demanded plans for an authentic, inhabitable city, highlighting the need to reclaim Venice’s “streets and squares”^100^. In June 2020, during the first months of the Covid-19 pandemic, a demonstration with the slogan “*Venezia FuTuristica*” (“was touristic” as well as “futuristic”) yet again pointed out the subordination of politics to industrial interests. At the same time, however, they used this moment, with its conspicuous absence of tourists, to raise questions about the post-pandemic development. By creating a human chain embracing the city, the protesters once again used their bodily presence to communicate their vision for a sustainable urban future.

## Transnational Relations and Circulation of Knowledge

Political mobilization against cruise ships is a transnational phenomenon that reaches beyond Europe, as the long-standing connection of the committee to the protest network in Key West shows.^22^ European protests took and take place in Barcelona, Marseille and Dubrovnik, which have become popular tourist destinations over the last 30 years (Perucic & Puh 2012), as well as in Kiel, which has long been a central shipping hub. Global developments and digital tools shape not only the content of protests but also their actor constellations, organizational forms, and effects (Daphi & Deitelhoff 2017: 306). Social media allows activists to provide each other with moral support while visualization tools allow the committee to portray the ship as an intruder that threatens both activists and residents alike. Aside from their local usages, the committee’s framings have also been shared by other groups.

A heterogeneous social and political structure is a common feature of current transnational protest movements, and this hybridity signals solidarity in the establishment of “networks of trust” (Della Porta/Tarrow 2005: 230). An explicitly non-identitarian framing is an important feature of such movements, since it allows for supportive and encouraging forms of transversal networking.^23^ Joint appearances both online and offline have a strong symbolic character and raise public awareness. What unites the members of the protest network against big ships and infrastructures is their ability to give a public face to an otherwise intangible phenomenon. The symbol of the large ship allows a diverse number of protest groups to call attention to the power imbalances created by dislocated and deterritorialized global economies, and to the effects they have on local practices, relations, and spaces.

Current debates surrounding the cruise industry in other cities also place contemporary issues in their historical contexts. In Hamburg, for example, the artist collective *Geheimagentur* (“secret agency”) carried out a project entitled “A Cruise Terminal” in 2015. Motivated by the opening of the third cruise terminal in the city, it led to discussions about who owns the “Right-to-the-Sea”, as well the prospect of “another cruise and another port” (Geheimagentur 2021).^24^ In doing so, the participants positioned themselves in opposition to the hegemonic discourse, since the arrival of cruise ships in Hamburg tends to be an important event that attracts thousands of people. Their aim was to put other kind of mobilities in perspective. Furthermore, the deepening of the river Elbe, which connects the city with the North Sea, has led to debates over ecological impacts since decades. Projects like the one led by *Geheimagentur* intend to bring untouched issues into focus. An intense transnational European collaboration has been carried out by the *Naturschutzbund Deutschland *(NABU), a German conservation group. NABU initiated a pilot study of atmospheric nitrogen oxide levels in the Mediterranean Sea, with the goal of analyzing the air quality in port cities. This campaign included experts and researchers from several European countries. Together with the Italian environmental association Citizens for the Air (*Cittadini per l’Aria*), the *Comitato No Grandi Navi, AmbienteVenezia*, and the group *We are here Venice* came together in a transnational campaign to “Let the Mediterranean breathe” (*Facciamo respirare il mediterraneo*). In March 2017, with the “Declaration of Rome,” European environmentalists called for Emission Control Areas (ECAs, special shipping zones defined by the International Maritime Organization) for the Mediterranean (Cittadini per l’Aria 2021).^25^ As of now, it is mostly ports located in Northern Europe which belong to such zones. The central demand of the declaration was the inclusion of all European seas in ECAs, which could be achieved by 2025.

Doreen Massey calls attention to the intersections which originate from networks of social relations: “[…] what gives a place its specificity is […] the fact that it is constructed out of a particular constellation of social relations, meeting and weaving together at a particular locus” (1991: 28). Although located or active in different places, the members of the network are connected on a normative level with common ways of thinking and visions, but also on a practical level, working together, acquiring knowledge, circulating and publishing it. Besides, activists and civic campaigners point to the responsibility of local and national governments and increasingly highlight their “role as market actors” who have to carry out their “regulatory influence on the market” (Kryst/Zajak 2017: 63). They share the urge for innovative and social approaches, administration, and narratives, considering local peculiarities and characteristics, as the local is itself “restructured and reproduced in interaction with translocal influences” (Knecht 2010: 25).

## Producing Knowledge, Proposing the Future Rhythm

Infrastructural planning has been reshaping the topography of Venice and its lagoon for ages, affecting the footpaths and waterways that contribute to the identity of the city and its inhabitants. The example of Venice shows that cruise infrastructures are a fundamental component of social order. By connecting their spatial knowledge to contested infrastructures, the movement *Comitato No Grandi Navi* has become a considerable voice in local politics. It has also managed to turn the cruise ship into a symbol representing this process.

The *Comitato *generates counter- and extra-knowledge through the acquisition of expertise and the staging of protest performances. The committee uses this knowledge to create a counter-narrative that portrays the cruise ship as an intruder, highlighting the focus on profit and growth that characterizes political decision-making. The *Comitato* puts emphasis on the fact that expertise should not exclusively be bound to institutions in charge, and demonstrates the relevance of the everyday and spatial knowledge that a heterogenous group of residents can bring to bear on the issue. While local and national governments express their visions in policies, the protesters provide alternative perspectives. Their tacit and embodied knowledge turns them into legitimate speakers and actors. In addition, the residents fight against popular views of Venice as a tourist city by showing that its everyday life works by logics that stand in conflict with those of the cruise industry. They provide images and formulate narratives of a city in which local life takes center stage. Through their spatial actions, their explicit knowledge, and their wide range of transversal and transnational interactions, the group influences local, national, and European developments in governance. Under these circumstances, the relational approach to infrastructures becomes visible and manifests itself in conscious local approaches and activities.

Their counter-narrative helps to further disseminate their own expertise. The committee managed to establish the cruise ship as a symbol representing the negotiation of social conditions. At the same time, it functions as a link to actors elsewhere. The ship has a mediating role between the perspectives of different actors because it enables the creation of ever new meanings and allows for the establishment of transnational connections. In communicating their counter-narrative to the international public, the committee uses a number of different counter-symbols, ranging from their bodies to smaller and more traditional boats, manifesting the everyday rhythm and the use and knowledge of urban space. In their aim to keep the local rhythm noticeable, they render this position visible through their appropriation of symbolic spaces and places. Virtual spaces, too, have become integral in giving visibility to topics that people feel need to be shared and discussed. On Facebook, for example, the page “Venetians” depicts the city as seen by its actual inhabitants. In this way, both offline and online projects show the agency of the city’s residents and portray it in ways usually not included in the clichés offered by the tourism industry. When large ships were banned from passing San Marco in August 2021, the *Comitato No Grandi Navi* achieved its first major success. They pronounced that “there was no going back” (“indietro non si torna”), inviting others to join them “not only to support our struggle, but to say all together that it can’t go back to the way it was before, with the monoculture of tourism, the selling off of public property, cementing and land consumption.” (NoGrandiNavi 2021).

However, this does not mean that the struggle between activists and the cruise industry is over. As far as infrastructures and the communities they affect are concerned, it is important to examine the issue of agency. Who is legitimized to speak on behalf of whom? And who remains voiceless? In cruise cities, tourism, work, and migration interact. What meaning does the cruise ship have for those who work in industries linked to the manufacture of vessels? What types of transregional and transnational alliances might be built? These are questions that deserve further research.

During the Covid-19 pandemic, perspectives changed yet again, and new voices spoke out in support of tourism. The pandemic brought the industry to a standstill for several months. While the cruise ship had divided Venetians into supporters and opponents before, new alliances like Yes Big Ships (*Si Grandi Navi*) were founded to strengthen the voice of those employed in the suddenly suffering industry. This once again has placed the symbol of the large ship in a new context. The pandemic multiplied the heterogeneous perspectives on infrastructural futures and has demonstrated once more the interplay between local and global structures.

## Footnotes

1 Within the last decade, several publications have highlighted the ecological, social and cultural consequences of cruise tourism in urban settings. All Italian and German quotes have been translated into English by the author.

2 No Grandi Navi 2021, http://www.nograndinavi.it/, 16.2.2022.

3 Das Venedig Prinzip, 2012, filmtank, directed by Andreas Pichler.

4 I would like to thank the two anonymous reviewers as well as the guest editors, whose valuable feedback contributed to my argument in this paper.

5 Horowitz, Jason: Too Big to Sail? A Plan for Venice’s Cruise-Ship Armada. *The New York Times*. 8^th^ November 2017. https://www.nytimes.com/2017/11/08/world/europe/venice-cruise-ships.html, 16.2.2022. Robbins, Neal. E.: Deep trouble: can Venice hold back the tide? *The Guardian.* 10^th^ December 2019. Mailing list of the *Comitato No Grandi Navi*.

6 Since the maritime economy has a long history not only in the Veneto region but also in neighbouring Friuli-Venezia Giulia, Monfalcone, a second production site of cruise ships, will also be examined in the further course of my research. The effects of the coexistence of cruise tourism and shipbuilding will be examined in both locations.

7 Interview with E. V., 16.7.2020, and Interview with S. M., 26.10.2019.

8 *AmbienteVenezia*, Dicembre 2020: La storia infinita delle grandi navi da crociera a Venezia e in Laguna. Mailing list of the *Comitato No Grandi Navi*.

9 Ahoi 2019: Das Kreuzfahrten-Magazin von Ruefa (3), Wien: Ruefa GmbH.

10 Attempts to raise awareness of the industry located on the mainland and to demystify Venice started increasingly after the flooding of 1966.

11 “Signor Rossi in Venice”, 1974, https://www.youtube.com/watch?v=4Vq4Iyc-8z0, 16.2.2022.

12 The last referendum to separate the lagoon city from the mainland, which failed, was held in December 2019.

13 No Grandi Navi, 2.6.2021: *Manifestazione contro le navi da crociera a Venezia, *https://www.globalproject.info/it/in_movimento/manifestazione-contro-le-navi-da-crociera-a-venezia/23507, 16.2.2022.

14 Besides Marghera, there are other spots in the lagoon where the ships currently land. Since there have already been a number of incidents in Marghera, an increasing number of vessels is heading for the cities of Monfalcone and, above all, Trieste.

15 Fiom Venezia 2018: *No alle grandi navi a Marghera, *https://www.fiom-cgil.it/net/index.php/fiom-veneto/5836-fiom-venezia-no-alle-grandi-navi-a-marghera, 16.2.2022.

16 La Nuova, 1.8.2021, *Da 15 anni diciamo no alle grandi navi. La prossima protesta? Al canale dei petroli, *19.

17 The constellation on site has changed considerably in the meantime, and the future use of the terminals (e.g. as landing place for maxi yachts) are up for debate. The resolution from August 2021 has resulted in several new developments and infrastructural debates that could not be addressed in detail in the contribution as it was completed before.

18 Alongside the protests, NOGrandiNavi held a self-managed referendum in June 2017 and collected over 18,000 signatures in one day calling for a different policy on dealing with cruise ships, see http://www.nograndinavi.it/ecco-i-risultati-del-referendum-autogestito/, 16.2.2022.

19 No Grandi Navi 2021, http://www.nograndinavi.it/appeal-to-european-movements-for-the-defence-of-territories-environmental-justice-and-democracy/, 16.2.2022.

20 At the local level, the main conflict emerged around the reconstruction of the main station in Stuttgart. The debates are mainly concerned with the costs and benefits of the project, as well as with environmental issues and the political execution of the process.

21 The abbreviation Tav stands for “Treno ad Alta Velocità”, high speed train. The No Tav movement fights against high-speed railway infrastructures between Turin and Lyon.

100 Venezia Today/Editorial office: „This is not Veniceland!“ Centinaia a Venezia contro le grandi navi e per la residenzialità. Venezia Today. 10th June 2018. http://www.veneziatoday.it/cronaca/corteo-marcia-dignita-oggi-venezia-no-navi-10-giugno-2018.html, 16.2.2022.

22 An Italian-American citizen established the contact between the protesters in 2013. The youtube video gives some impressions of the current Key West Cruise Ship Protest: https://www.youtube.com/watch?v=vZzYTnN52-Y, 16.2.2022.

23 The Globalproject platform documents the transnational networking of protesters, https://www.globalproject.info/, 16.2.2022.

24 Geheimagentur, https://www.geheimagentur.net/projekte/ein-kreuzfahrtterminal-3/, 16.2.2022.

25 Cittadini per l’aria 2021, Facciamo respirare il Mediterraneo, https://www.cittadiniperlaria.org/facciamo-respirare-il-mediterraneo/, 16.2.2022.
